# Childhood maltreatment and lead levels in middle adulthood: A prospective examination of the roles of individual socio-economic and neighborhood characteristics

**DOI:** 10.1371/journal.pone.0240683

**Published:** 2020-11-24

**Authors:** Anthony Carpi, Valentina Nikulina, Xuechen Li, Cathy Spatz Widom

**Affiliations:** 1 Department of Sciences, John Jay College, New York City, New York, United States of America; 2 Psychology Department, Queens College, Queens, New York, United States of America; 3 The Graduate Center, City University of New York, New York City, New York, United States of America; 4 Psychology Department, John Jay College, New York City, New York, United States of America; Graduate School of Public Health and Health Policy, City University of New York, UNITED STATES

## Abstract

**Background:**

Lead is a common environmental hazard because of its past use as an additive to gasoline and household paint. Some evidence suggests that children with histories of child abuse and neglect are at elevated risk for residence in communities and households with less desirable characteristics and high levels of exposure to environmental hazards and toxins.

**Objectives:**

To understand whether childhood maltreatment leads to higher levels of household dust lead and blood lead in adulthood and the extent to which characteristics of a person’s physical environment or individual level socio-economic status (SES) (based on unemployment, poverty, and receipt of public assistance) contribute to understanding the relationship.

**Methods:**

A large prospective cohort design study in which abused and neglected children (ages 0–11) were matched with non-maltreated children and assessed in adulthood. Objective and subjective neighborhood characteristics were assessed at approximate age 40 and household dust lead (cleaned and less often cleaned) and blood lead levels were measured at age 41. Blood was collected through venipuncture by a registered nurse as part of a medical status exam.

**Results:**

Childhood maltreatment predicted higher levels of dust lead in less often cleaned household places, residence in worse neighborhoods defined by objective (census tract data) and subjective (reports of physical disorder and lack of social cohesion and control), and higher levels of poverty, receiving public assistance, and unemployment. Only objective neighborhood characteristics mediated the relationship between childhood maltreatment and dust lead level in adulthood. There were also significant paths from objective neighborhood disadvantage and individual level SES to higher levels of blood lead.

**Discussion:**

Thirty years after their childhood experiences, individuals with documented histories of childhood maltreatment are at higher risk for living in environments as adults with elevated lead levels that may impact other aspects of their lives and compromise their health.

## Introduction

More than 3.5 million children were referred for possible maltreatment in fiscal year 2017, the most recent statistics available [1]. Child maltreatment represents a major public health problem in the United States and abroad, with lasting impacts on health, social, and economic functioning [2–6]. Childhood maltreatment has been linked to increased risk of living in neighborhoods with less desirable characteristics [7, 8] and higher levels of economic disadvantage [9–11].

Over the past several decades, the literature that associates both housing and neighborhood characteristics with negative health outcomes has grown [12–14]. For example, in a paper on income inequality and its effect on morbidity, Soobader and LeClere [15] noted the substantial body of research which demonstrates that the physical and social characteristics of where people live make an independent contribution to health, rather than serving as a surrogate for individual level data [16–18]. Claudio, Tulton, Doucette, and Landrigan [19] reported a strong relationship between hospital admission rates for asthma and low median household income and speculated that poor housing conditions, environmental exposures (indoor allergens and passive cigarette smoke), and lack of access to preventive health care may have contributed to the higher rates of asthma in some of the neighborhoods they studied.

Environmental exposure to lead is one such housing variable that remains a major public health concern. Due to its widespread use in leaded gasoline, paint, and plumbing systems–lead is linked to housing characteristics [20, 21]. And lead exposure has been positively related to housing age, and inversely to housing value, median per capita income, and other socioeconomic factors [22]. However, research has found that the associations between lead and health and behavioral outcomes are also heavily confounded by low socioeconomic status [23, 24].

Lead is a particularly troublesome environmental pollutant as there is increasing evidence that lead is associated with negative health effects even at levels far below thresholds previously considered safe [25–28]. The adverse effects of lead, including learning and behavioral disorders (e.g., attention deficit disorder and attention deficit hyperactivity disorder), hearing impairment, decreased intelligence quotient, and decreased attention span, are particularly harmful in children and often become apparent during puberty—long after exposure. Elevated blood lead (PbB) concentrations are known to have detrimental effects on neuropsychological function in both children and occupational cohorts of men and women [29]. Some researchers have suggested that even relatively small increases in lead in the body are associated with poor attention, academic failure [30]. In a recent paper, Obeng-Gyasi et al. [31] examined cardiovascular-related clinical markers in adults exposed to lead and found that lead exposure had a profound effect on the cardiovascular system. Harari et al. [32] found that low-level lead exposure was associated with decreased kidney functioning.

Maltreated children are already at risk for learning problems, school failure, and other maladaptive behaviors [33–37]. Elevated PbB may place these children at further risk of cognitive and behavioral problems. Some evidence suggests that maltreated children are more likely to live in communities and households with high levels of exposure to environmental hazards and toxins and to have elevated levels of lead. In one study, Bithoney, Vandeven, and Ryan [38] reported that children suspected of having been physically abused had significantly higher levels of lead than a comparison group. Flaherty [39] examined the prevalence of lead poisoning in abused and neglected children (ages 6 months to 6 years) taken into protective custody in Cook County, Illinois, in 1992 and found that almost two-thirds (64.7%) of the children tested had elevated lead concentrations. The rates of elevated PbB in these Cook County children were 2–3 times higher than children in urban and rural areas and 30 times higher than children living in suburbs. However, whether maltreatment as a child represents a further disadvantage to individuals by increasing the likelihood that they are exposed to environmental hazards as adults remains a question.

### The present study

**Fig 1:**
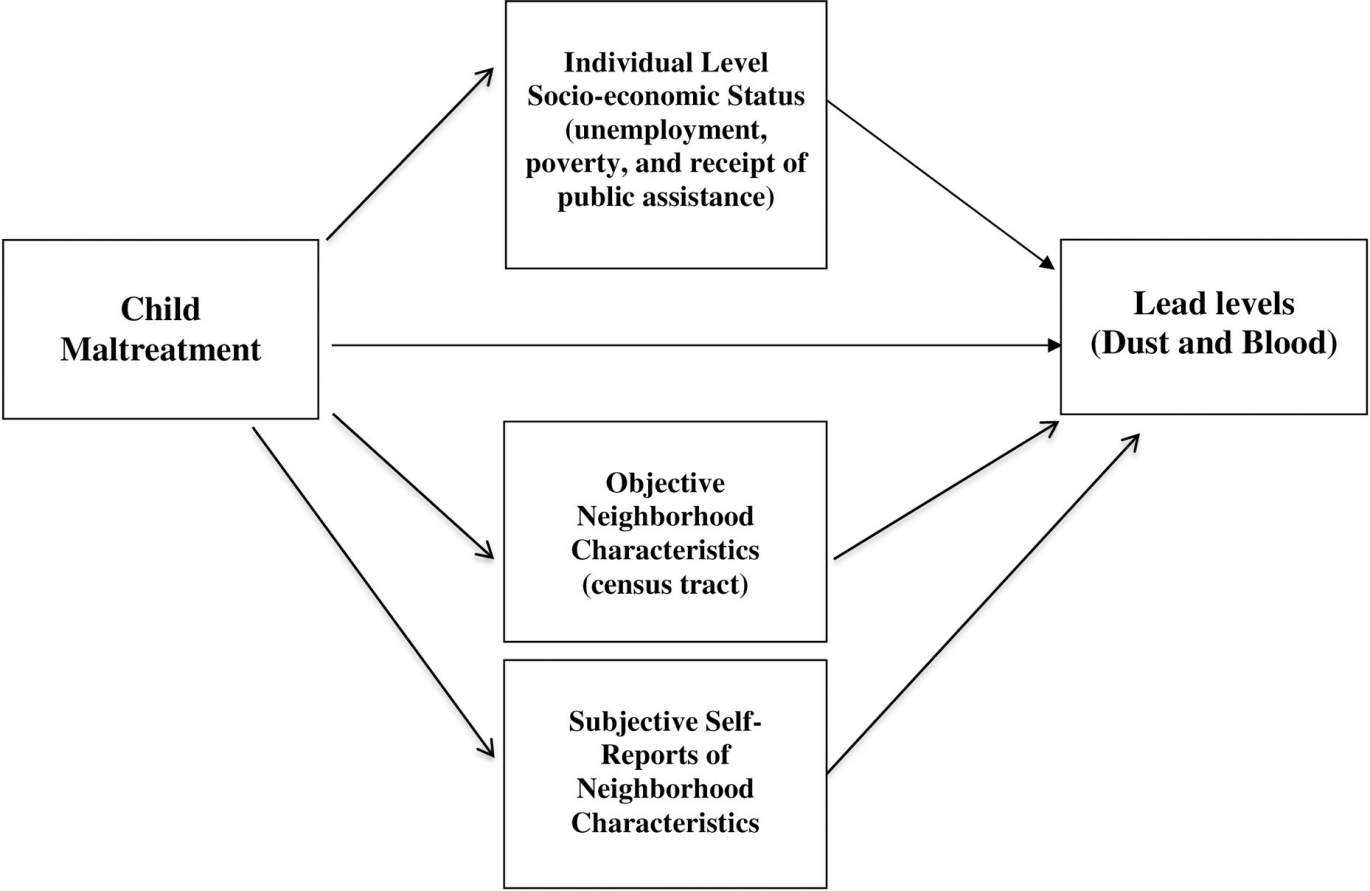
Hypothesized model showing a direct path from childhood maltreatment to elevated dust and blood lead levels in middle adulthood and three different pathways (or mechanisms) that may explain these relationships. One potential pathway from childhood maltreatment to higher lead levels in adulthood is through residence in worse neighborhoods, based on objective census tract data and a second is through neighborhood characteristics based on subjective self-reports. The third hypothesized pathway is through a person’s individual level socio-economic status (unemployment, poverty, and receipt of public assistance).

This study seeks to understand the relationship between childhood maltreatment and exposure to household lead pollution as an adult, to determine whether maltreated children are at increased risk for living in households that may place them at risk for higher blood and dust lead levels, and the extent to which a person’s individual socio-economic characteristics or characteristics of the physical environment contribute to understanding the hypothesized relationship. **Fig 1** shows a hypothesized direct path from child maltreatment to elevated dust and blood lead levels in middle adulthood, but also includes three different pathways (or mechanisms) that may explain this increased risk. One explanation for this relationship might be through residence in disadvantaged neighborhoods with poor housing stock. Because maltreated children are at risk for living in neighborhoods with greater economic disadvantage, we examined whether childhood maltreatment might lead to higher lead levels through residence in objectively defined worse neighborhoods based on census tract. Second, we considered whether childhood maltreatment leads to high lead levels through residence in neighborhoods defined by subjective self-reports of neighborhood qualities. Third and alternatively, because maltreated children are at increased risk for higher rates of unemployment, poverty, and receipt of public assistance—individual markers of socio-economic status (SES) we tested whether these individual level SES characteristics explained the relationship between childhood maltreatment and elevated levels of blood and dust lead in adulthood.

This is the first prospective study of documented cases of childhood maltreatment and matched controls that were followed up into middle adulthood and assessed for blood lead as adults. Their adult residences were also assessed for dust lead at the same time. We then examined the relationship between child maltreatment and dust/blood lead levels and whether neighborhood or individual level characteristics explained or mediated the relationship. This study offers several advantages. First, we used unambiguous definitions of child maltreatment, including physical and sexual abuse and neglect. Second, we used documented cases of childhood maltreatment to minimize potential problems with reliance on retrospective self-reports. Third, unlike most cross-sectional studies of childhood maltreatment, we used a prospective matched cohort design, thereby providing an appropriate comparison group and assessment of the correct temporal sequence of events. Fourth, we used measured outcomes through blood test and dust collection, rather than self-reports of medical problems. Fifth, we traced development beyond adolescence and young adulthood to middle adulthood. Finally, the large heterogeneous sample includes men and women and is varied in terms of race/ethnicity.

## Methods

These data were collected as part of a large prospective cohort design study [40, 41] in which abused and neglected children were matched with non-abused and non-neglected children and followed into adulthood. Because of the matching procedure, the participants are assumed to differ only in the risk factor, that is, having experienced childhood sexual or physical abuse or neglect. Because it is not possible to assign participants randomly to groups, the assumption of equivalency for the groups is an approximation. The control group may also differ from the abused and neglected individuals on other variables nested with abuse or neglect. For complete details of the study design and participant selection criteria, see Widom [42].

The original sample of abused and neglected children (*N* = 908) comprised all substantiated cases of childhood physical and sexual abuse and neglect processed from 1967 to 1971 in the county juvenile (family) or adult criminal courts of a Midwestern metropolitan area. Cases of abuse and neglect were restricted to children 11 years of age or younger at the time of the incident.

A critical element of the design involved the selection of a matched control group. Controls were matched with the sample on the basis of age, sex, race/ethnicity, and approximate family social class during the time period under study. Matching for approximate family social class was important in this study because it is theoretically plausible that any relationship between child abuse and neglect and subsequent outcomes may be confounded with or explained by social class differences [43, 44]. It is difficult to match exactly for social class because higher income families could live in lower social class neighborhoods and vice versa. The matching procedure used here is based on a broad definition of social class that includes neighborhoods in which children were reared and schools they attended. Similar procedures, with neighborhood school matches, have been used in studies of people with schizophrenia [45] to match approximately for social class.

Where possible, two matches were found to allow for loss of control group members: that is, individuals who were initially selected for the control group who were reported in the official abuse and neglect files were eliminated and replaced, where possible, with a second matched control subject. Any control child with an official record of abuse or neglect was eliminated, regardless of whether the record was before or after the period of the study. This occurred in 11 cases.

To accomplish the matching, the control sample, like the abuse and neglect sample, was divided into two groups, those under and those of school age at the time of the abuse or neglect incident. Using county birth record information, children *under school age* were matched with children of the same sex, race, date of birth (+/ 1 week), and hospital of birth. Of the 319 abused and neglected cases, matches were found for 229 (72%) of the group. Children *of school age* were matched as closely as possible by sex, race, date of birth (+/- six months), and class in the elementary school system during 1967 through 1971. Records of more than 100 elementary schools for the same time period were used to find matches. Busing was not operational at this time, and students in elementary schools in this county were from small, socioeconomically homogeneous neighborhoods. Matches were never made with students from another school, although it was sometimes necessary to select students from different classes or even different grades in the same school. Where an abused or neglected child had been held back a grade, resulting in a discrepancy between the child’s age and grade, the match was made on the basis of age. Where a child had attended special education classes during the period, attempts were made to include matches from such classes. Of the 589 school-age children in the abuse and neglect group, we found matches for 438 or 74% of the abused and neglected children.

Non-matches occurred for a number of reasons. In the case of birth records, they occurred if the abused or neglected child was born outside the county or state, or if information about the date of birth was missing. In the case of school records, non-matches occurred because the elementary school had closed during the years since 1971 and class registers were consequently not available, or because schools had been primarily uniracial (they were not necessarily integrated at the time) and a same race match could not be found. We reanalyzed earlier findings using only matched pairs, and the results did not change with the smaller sample size [44, 46].

In the initial phase of the study, we compared the abused and neglected children to the matched comparison group on juvenile and adult criminal arrest records (*N* = 1,575) [44]. A second phase involved tracking, locating, and interviewing both groups during 1989–1995, approximately 22 years after the abuse or neglect (*N* = 1,196). We conducted subsequent follow-up interviews in 2000–2002 (*N* = 896) and 2003–2005 (*N* = 807) that included a comprehensive health interview, physical health measures, blood collection through venipuncture, dust collection, and other questionnaires and assessment instruments.

Although there was attrition associated with death, refusals, and our inability to locate individuals over the various waves of the study, the composition of the sample at the four time points has remained about the same (see **Table 1**). The abuse and neglect group represented 56%–58% at each period; non-Hispanic Whites comprised 62%–66%; and males comprised 48%–51% of the samples. There were no significant differences across the samples on these variables or in mean age across the four phases of the study. Analyses were restricted to participants who identified as Black and White (a small group of Hispanics was excluded due to their relatively small number). The current total sample (*N* = 631) was on average 41.3 years (SD = 3.5, range 32.0–49.0), 57.2% women, and 61.6% White, non-Hispanic. The sample was predominantly from the lower end of the socioeconomic spectrum: 60.0% completed high school, 54.9% held unskilled or semiskilled jobs, and only 13.7% held semiprofessional or professional jobs [47]. This characteristic limits the extent of generalizing to children from middle and upper socio-economic status (SES) families. There were no significant differences between participants in this wave of the study who gave blood or whose home was tested for dust lead and the participants who did not in terms of age (*p* = .522), sex (*p* = .097), race (*p* = .490), and abuse/neglect status (*p* = .570).

**Table 1:** Characteristics of the study sample at different waves of data collection.

	Records	Interviews		
		1	2	3
Year of data collection		1989–1995	2000–2002	2003–2005
N	1575	1196	896	808
CHARACTERISTICS				
Sex (% male)	49.3	51.3	49.0	47.3
White (%)	66.2	62.9	62.2	60.4
Black (%)	32.6	34.9	35.2	37.0
Other (%)	1.2	2.2	2.6	2.6
Hispanic (%)	0.3	3.8	4.0	4.0
Abuse/neglect (%)	57.7	56.5	55.8	56.8
Mean age at petition (SD)	6.4 (3.3)	6.3 (3.3)	6.2 (3.3)	6.3 (3.3)
Mean age at Interview (SD)		29.2 (3.8)	39.5 (3.5)	41.2 (3.5)

### Procedures

Participants were interviewed in-person in their homes or other quiet locations of their choice. Interviewers were blind to the purpose of the study and to the inclusion of an abuse and neglect group. Participants were also blind to the purpose of the study and were told that they had been selected to participate as part of a large group of individuals who grew up in that area during the late 1960s and early 1970s. City University of New York Institutional Review Board approval (Protocol #: 2015–0133) was obtained for the procedures involved in this study and those who participated signed a consent form acknowledging that they understood the conditions of their participation and that they were participating voluntarily. For those with limited reading ability, the consent form was read aloud to the participant and, if necessary, explained verbally.

### Measures

#### Independent variable

*Child abuse and neglect*. We assessed childhood physical and sexual abuse and neglect through review of official records processed during 1967–1971. Physical abuse cases included injuries such as bruises, welts, burns, abrasions, lacerations, wounds, cuts, bone and skull fractures, and other evidence of physical injury. Sexual abuse cases include felony sexual assault, fondling or touching, sodomy, incest, and rape. Neglect cases reflected a judgment that the parents’ deficiencies in childcare were beyond those found acceptable by community and professional standards at the time and represented extreme failure to provide adequate food, clothing, shelter, and medical attention to children.

#### Potential mediators

*Neighborhood characteristics*. Subjective and objective neighborhood characteristics were assessed during the second interview when participants were mean age 40. Assessment of subjective neighborhood characteristics was based on participants’ responses to questions about characteristics of their current neighborhood. *Physical disorder* [48] was assessed with 12 self-report items asking about the extent of certain problems, such as graffiti, using a 4-point Likert scale (α = 0.93). *Social cohesion* [49] was measured using eight self-report items, such as "People here are willing to help their neighbors” on a 5-point Likert scale (α = 0.83). *Social control* [49] was assessed with 7 self-report items (e.g., "Someone was trying to sell drugs to your children in plain sight?") on a 4-point Likert scale (α = 0.90). Higher scores indicate greater physical disorder, lack of social cohesion and lack of social control.

For the objective measure of neighborhood economic disadvantage, participants’ addresses from the 2000–2002 interviews were geo-coded to 2000 census tract data that represented approximately 40 states across the US where they were living at that time [50]. Three variables were used to provide an objective indicator: (1) percentage of families below the poverty line, (2) percentage of families on public assistance, and (3) percentage of woman headed households. Each of these variables was first standardized and then summed to provide an index representing an objective measure of neighborhood economic disadvantage.

*Individual level socio-economic status*. Three indicators were used to assess an individual’s socio-economic status: (1) income—the sample was divided into two groups (lower and higher) using a cut point at the poverty line in the US in 2001(using HHS poverty guidelines, for a family of four the household income threshold of poverty level is $17,650); (2) receipt of public assistance—whether the person was receiving food stamps or Medicaid [125 (18.6%) were receiving food stamps and 149 (22.1%) were on Medicaid]; and (3) unemployment—participants were divided into those employed (and this included homemakers and those in school) and not employed (including disabled).

#### Outcome variables

*Dust lead*. The dust was collected in a protocol adapted from ASTM International standard E1728-02, “Standard Practice for Collection of Settled Dust Samples Using Wipe Sampling Methods for Subsequent Lead Determination.” Field interviewers were given detailed instructions about choosing two specific areas in the home to wipe, with hard surfaces and not ones that had carpeting: (1) the top of the refrigerator for one area (area less often cleaned) and (2) the kitchen or bathroom floor or kitchen table or countertop for the second (area more often cleaned). Interviewers used gloves to mark the area to be sampled without disturbing dust and then taped the surface edge of the template. Using individually packaged wipes, the interviewers wiped the area three times with the same cloth. After collecting the dust, wipes were folded several times and then inserted in sample containers. Information was recorded in the interview record book and, for each included date, number identifying the sample (1 for refrigerator and 2 for kitchen table or countertop), short description of the site, lot number of wipes, and dimensions of the area sampled (actually wiped) in centimeters. Similar sample and case information was recorded on the sample container to prepare it for shipping. As a quality control measure, on every 5^th^ interview and every 15th thereafter, the interviewer was instructed to create a field blank, following the procedures without wiping anything and but inserting blank wipes into container and sending for processing. Containers were sent to Modern Industries, Free Col laboratory, Medville, Pennsylvania. Sample preparation involved sonication for metals following EPA method 600/R-93/200, and lead was analyzed using Graphite Furnace Atomic Absorption spectroscopy. Dust lead outliers (>50 ug/mm^2^) were replaced with 50 ug/mm^2^.

*Blood lead*. A licensed registered nurse performed the medical status examination in the participant’s home or another quiet location of the participant’s choice. Nurses collected 45 milliliters of blood for all the medical screens and assays. Nurses observed universal precautions during all draws, using standard venipuncture procedures, through a single venipuncture using a small gauge butterfly needle and multiple draw adapter. Blood was wrapped and shipped overnight to University Hospital, Newark, New Jersey. Nurses also labeled and completed a chain-of-custody form for each person’s sample and placed it in the transport box with the tubes. The University Hospital Pathology Laboratory conducted routine blood tests. After removing the 116 people who did not give blood, the sample was 557 people. Blood levels ranged from 1–18 ug/dl and were treated as continuous variables.

### Statistical analysis

The first step in the data analysis was to conduct basic descriptive statistics to examine characteristics of the maltreated and control groups using T-tests for continuous variables and Pearson’s chi-square tests for categorical variables. We then conducted exploratory factor analyses (EFA) to determine whether the neighborhood and individual level SES variables would converge as latent constructs. All potential mediators were included. We used oblique rotation and Ordinary Least Squares (OLS) to find the minimum residual (MINRES) solution. The results indicated that the objective and subjective neighborhood characteristics loaded onto one factor and household income, receiving public assistance and unemployment on a second one. Although objective and subjective neighborhood characteristics are highly related, they are not necessarily indicative of one another. Based on prior theory and research distinguishing between objective and subjective neighborhood characteristics [49], we consider the two sets of neighborhood variables separately. Objective neighborhood characteristics are based on census data. A latent construct representing subjective neighborhood characteristics includes physical disorder (loading = 0.705), lack of cohesion (loading = 0.594), and lack of social control (loading = 0.411). A latent construct for individual level SES includes household income (loading = 0.822), receiving public assistance (loading = 0.575), and unemployment (loading = 0.370).

The next step in the analysis involved a series of structural equation models (SEM) to test for mediation. In this study, we used lavaan (latent variable analysis) with R to test for mediation (https://lavaan.ugent.be/tutorial/mediation.html). Similar to classical mediation, the analysis involved the three variables (Y is the dependent variable, X is the predictor, and M is the mediator) and we fit a path analysis model that included the direct of X on Y and the indirect effect of X on Y via M. The standard errors for these parameters are computed by using the so-called Delta method. For each model, childhood maltreatment was used as the independent variable and dust lead or blood lead as the dependent variable. Neighborhood (objective and subjective) and individual level SES were examined in separate models. These analyses were followed by two additional SEM models (one for dust lead and one for blood lead level) that incorporated both the two latent constructs (subjective neighborhood and individual level SES) and objective neighborhood variables as mediators in the models. All models controlled for age, sex, and race. Because of missing data, full information maximum likelihood (FIML) was used to estimate the path coefficients, which uses information from all available data. Fit indices were examined to determine goodness of fit [chi square = *p* > 0.05; CFI (comparative fit index) > 0.95; TLI (Tucker-Lewis Index) > 0.95; RMSEA (root mean square error of approximation) ≤ 0.05)]. Standardized coefficients (β) are reported for all models. All analyses were run in R [version 3.5.2, R-package lavaan (version 0.6–3), psych (version 1.7.2)].

## Results

**Table 2** presents descriptive characteristics of the study sample. Simple bivariate statistics show that there were no differences in demographic characteristics of the two groups (maltreated and controls). However, as expected, the two groups differed significantly in terms of objective and subjective neighborhood characteristics (with the exception of social control) and individual level SES indicators. Individuals with documented histories of childhood maltreatment were living in worse neighborhoods in adulthood and had lower individual SES levels (that is, lower household income and higher levels of unemployment and receipt of public assistance). Maltreated and control participants did not differ significantly in terms of blood lead level or dust lead level based on the area more often cleaned, but differed significantly in dust lead level in the area less often cleaned (t = 2.29, *p* = 0.024).

**Table 2:** Descriptive characteristics of the study sample and by maltreatment status.

	Total	Control	Maltreated	*p*
	(N = 631)	(N = 280)	(N = 351)	
	N (%)	N (%)	N (%)	
Sex, female	361 (57.2%)	155 (55.4%)	206 (58.7%)	0.448
Race				
White, not Hispanic	389 (61.6%)	168 (60%)	221 (63%)	0.498
Black	242 (38.4%)	212 (40%)	130 (37%)	
	**M (SD)**	**M (SD)**	**M (SD)**	
Objective neighborhood–census tract	0.6 (2.8)	0.4 (3.0)	0.8 (2.7)	0.046
Subjective neighborhood characteristics				
Physical disorder	1.7 (0.8)	1.6 (0.7)	1.7 (0.9)	0.006
Lack of social cohesion	2.6 (0.8)	2.5 (0.7)	2.7 (0.8)	< .001
Lack of social control	1.8 (0.8)	1.8 (0.7)	1.8 (0.8)	0.156
Individual level socio-economic status				
Household income (poverty)	178 (28.2%)	50 (17.9%)	28 (36.5%)	< .001
Public assistance	147 (23.3%)	44 (15.7%)	103 (29.3%)	< .001
Unemployed	113 (17.9%)	39 (13.9%)	74 (21.1%)	0.026
	**M (SD) N**	**M (SD) N**	**M (SD) N**	
Blood (ug/dL)	3.3 (1.9) 533	3.4 (2.2) 223	3.3 (1.7) 310	0.526
Dust, less cleaned (μg/mm^2^)	2.0 (6.0) 612	1.4 (3.6) 269	2.5 (7.4) 343	0.024
Dust, more cleaned (ʼg/mm^2^)	1.1 (3.4) 596	0.9 (3.7) 264	1.3 (3.1) 332	0.111
Age when dust sample collected	41.3 (3.5)	41.2 (3.6)	41.3 (3.5)	0.500

Note: Census tract based on multiple indicators and then standardized.

**Table 3** shows the results of three separate structural equation models (SEM) testing whether child maltreatment predicts dust lead levels in less often cleaned places through each of the three potential mediators (objective and subjective neighborhood characteristics and individual level SES), controlling for age, sex and race. The first line in shows that there is a significant total effect of childhood maltreatment predicting higher dust lead levels in areas less often cleaned (total effects: β = 0.09, *SE* = 0.04, *p* = 0.03). The second section of the table focuses on the objective (census) neighborhood characteristics and whether these neighborhood characteristics mediate the relationship between child maltreatment and dust lead level in less often cleaned places in adulthood. These results indicate that there were significant paths from childhood maltreatment to dust lead (less cleaned) (β = 0.08, *SE* = 0.04, *p* = 0.04) and to objective (census based) neighborhood characteristics (β = 0.08, *SE* = 0.04, *p* = 0.05) and from objective neighborhood characteristics to dust lead level (β = 0.10, *SE* = 0.04, *p* = 0.02). The indirect path from childhood maltreatment to dust lead level via objective neighborhood characteristics was not significant (β = 0.01, *SE* = 0.01, *p* = 0.13).

**Table 3:** Individual structural equation models predicting levels of dust lead in less often cleaned places.

						Model Statistics				
Paths	β	SE	CI lower	CI upper	*p*	χ^2^	*p*	CFI	TLI	RMSEA
Total effects: CM -> Dust lead, less cleaned	0.09	0.04	0.01	0.17	**0.03**					
Direct: CM -> Dust lead, less cleaned	0.08	0.04	0.00	0.16	**0.04**	123.3	<0.01	0.091	0.000	0.253
CM -> objective neighborhood (census)	0.08	0.04	0.00	0.16	**0.05**					
Objective neighborhood -> dust lead	0.10	0.04	0.01	0.19	**0.02**					
Indirect: CM -> objective -> dust lead	0.01	0.01	0.00	0.02	0.13					
Direct: CM -> Dust lead, less cleaned	0.08	0.04	0.00	0.16	**0.05**	28.82	<0.01	0.928	0.877	0.044
CM -> subjective neighborhood	0.18	0.05	0.08	0.27	**0.00**					
Subjective neighborhood -> dust lead	0.06	0.05	-0.05	0.16	0.29					
Indirect: CM -> subjective -> dust lead	0.01	0.01	-0.01	0.03	0.31					
Direct: CM -> Dust lead, less cleaned	0.09	0.04	0.01	0.17	**0.03**	57.39	<0.01	0.847	0.742	0.074
CM -> individual level SES	0.27	0.04	0.18	0.35	**0.00**					
Individual SES -> dust lead, less cleaned	0.00	0.05	-0.10	0.10	0.97					
Indirect: CM -> SES -> dust lead	0.00	0.01	-0.03	0.03	0.97					

Notes: β = standardized change of dust lead level with one unit change in independent variable; SE = standard error; CI = 95% confidence interval; χ ^2^ = critical ratio chi square; degrees of freedom for chi-square test = 3, 13, 13; CFI = comparative fit index; TLI = Tucker-Lewis index; RMSEA = root mean square error of approximation; CM = child maltreatment. Objective neighborhood is measured based on census tract characteristics. All models in the table controlled for age, sex and race.

The third section of **Table 3** focuses on the potential role of subjective neighborhood characteristics as a mediator between childhood maltreatment and dust lead levels (in areas less often cleaned) in adulthood. Here, there are significant paths from childhood maltreatment to dust lead level and from childhood maltreatment to subject neighborhood characteristics (β = 0.18, *SE* = 0.05, *p* = 0.00), but not from subjective neighborhood characteristics to dust lead. Again, the indirect effect was not significant. **Table 3** also shows similar results for individual level SES. Childhood maltreatment predicts dust lead level (in areas less often cleaned) and childhood maltreatment predicts individual level SES in adulthood, but individual level SES in adulthood does not predict dust lead nor is there a significant indirect path.

**Table 4** shows the results of separate SEM models testing whether childhood maltreatment predicts blood lead levels in adulthood through the potential mediators of objective and subjective neighborhood characteristics and individual level SES, while controlling for age, sex and race. For blood lead levels, the first line in **Table 4** shows that the overall and total effect was not significant. However, in the next section of this table, the results show a significant path from child maltreatment to objective neighborhood characteristics as seen earlier (β = 0.08, *SE* = 0.04, *p* = 0.05) and there is a significant path from objective neighborhood characteristics to blood lead in adulthood (β = 0.19, *SE* = 0.04, *p* < 0.01). There is also a non-significant trend suggesting an indirect path from child maltreatment to blood lead level in adulthood via objective neighborhood characteristics (β = 0.02, *SE* = 0.01, *p* = 0.07).

**Table 4:** Individual structural equation models predicting blood lead levels.

						Model Statistics				
Paths	β	SE	CI lower	CI upper	*p*	χ^2^	*p*	CFI	TLI	RMSEA
Total effects: CM -> Blood lead	-0.02	0.04	-0.11	0.06	0.55					
Direct: CM -> Blood lead	-0.04	0.04	-0.12	0.04	0.33	122.4	<0.01	0.305	0.000	0.252
CM -> objective neighborhood (census)	0.08	0.04	0.00	0.16	**0.05**					
Objective neighborhood -> Blood lead	0.19	0.04	0.10	0.28	**<0.01**					
Indirect: CM -> objective -> Blood lead	0.02	0.01	0.00	0.03	**0.07**					
Direct: CM -> Blood lead	-0.03	0.04	-0.12	0.05	0.47	28.4	<0.01	0.937	0.894	0.044
CM -> Subjective neighborhood	0.18	0.05	0.08	0.28	**<0.01**					
Subjective neighborhood-> Blood lead	0.04	0.05	-0.06	0.15	0.42					
Indirect: CM -> subjective -> Blood lead	0.01	0.01	-0.01	0.03	0.43					
Direct: CM -> Blood lead	-0.05	0.04	-0.14	0.04	0.26	54.38	<0.01	0.871	0.781	0.033
CM -> Individual level SES	0.26	0.04	0.18	0.35	**<0.01**					
Individual level SES-> Blood lead	0.10	0.05	0.00	0.20	**0.04**					
Indirect: CM -> SES-> Blood lead	0.03	0.01	0.00	0.06	**0.06**					

Notes: β = standardized change of blood lead with one unit change in independent variable; SE = standard error; CI = 95% confidence interval; χ ^2^ = critical ratio chi square; degrees of freedom for chi-square test = 3, 13, 13; CFI = comparative fit index; TLI = Tucker-Lewis index; RMSEA = root mean square error of approximation; CM = child maltreatment. Objective neighborhood characteristics are based on census tract data. All models in the table controlled for age, sex and race.

The next section of **Table 4** focuses on subjective neighborhood characteristics. These results show a significant path from childhood maltreatment to subjective neighborhood characteristics (β = 0.18, *SE* = 0.05, *p* < 0.01), but no other significant paths in that model. In contrast, the final section of **Table 4** shows there are significant paths from child maltreatment to individual level SES (β = 0.26, *SE* = 0.04, *p* < 0.01) and from individual level SES to higher blood lead levels in adulthood (β = 0.10, *SE* = 0.05, *p* = 0.04). There is also a close to significant indirect path from childhood maltreatment to blood lead level in adulthood through individual level SES (β = 0.03, *SE* = 0.01, *p* = 0.06).

Two final SEM models were tested including the objective neighborhood characteristics as well as the latent constructs for subjective neighborhood and individual level SES predicting levels of dust lead in areas least cleaned (tables not shown but available from the authors upon request). With all variables in the model, although there was a significant direct path from child maltreatment to dust lead in a less often cleaned area (total effects: β = 0.09, *SE* = 0.04, *p* = 0.03, direct effect: β = 0.08, SE = 0.04, p = 0.05), there were no significant indirect effects and the model fit indicators showed poor fit. For blood lead level, the final model with all constructs and variables showed poor fit and no significant pathways.

## Discussion

The findings of the current study suggest that maltreatment experienced by children predicts the level of lead present in their home environment, three decades later, in middle adulthood. The differences in levels of lead in the homes of maltreated and control participants were observed in dust from the less often cleaned areas, not in areas more often cleaned, suggesting that participants did not differ in tidiness or cleanliness, but rather that there was more lead accumulation in their household environment. Particles from lead paint, contaminated soil, and other environmental hazards are part of home dust [51].

Our findings indicate that residence in neighborhoods in earlier adulthood that are characterized by objective indicators of (based on census data) help explain the association between childhood maltreatment and dust lead level later in middle adulthood. Prior research on a cross-sectional national dataset has found that factors associated with poverty, including age of the home, rental status, and smoking in the home, pose risk for high levels of dust lead in surfaces that are less often cleaned [52]. Here, longitudinal results demonstrate that neighborhood economic factors predict lead levels in their homes years later in adulthood.

One of the problems in attempts to understand the consequences of childhood maltreatment, particularly neglect, is the fact that many of these families live in poverty. These new findings are particularly meaningful because maltreated and control participants were matched on childhood circumstances and originated from similar communities. Therefore, the associations among childhood maltreatment, neighborhood disadvantage and dust lead are unlikely to be explained by levels of poverty in childhood. One of the implications of these findings is that the higher dust lead levels could increase the lead risk for their children.

These new findings show that lead exposure risk is not significantly greater in adults with histories of child maltreatment as ascertained by blood lead measurement (although there was a trend in that direction). When we designed this study, we were aware that blood is a transient marker and only reflects recent exposure to lead. However, we wanted to be comprehensive in our assessment to include both dust lead and blood lead. Based on the larger extant literature, the association we report here between objectively measured neighborhood disadvantage and blood level and the non-significant trend showing an indirect path from child maltreatment to blood lead level through objectively measured neighborhood characteristics suggests that housing stock for these individuals may be problematic and particularly problematic for their offspring. It is possible that offspring have higher blood lead levels that could increase levels of longer-term storage of lead, such as bone. Future research should investigate bone-lead levels in adults with histories of childhood maltreatment and blood-lead levels in their offspring.

Future research needs to examine other explanations for these relationships. Lower levels of academic achievement [53] and economic productivity [2], elevated psychopathology [43, 46, 54], higher levels of crime [55] and risk behaviors among maltreated children in adolescence and young adulthood may put them at risk for residence in worse neighborhoods [8] and result in higher levels of lead in their homes and apartments. Prior research has found that adults with histories of childhood maltreatment are at higher risk for poor health outcomes [3, 6, 56–58], including cardiovascular, endocrine and pulmonary systems, compared to demographically matched adults without such histories. It is also possible that there are neuropsychological or immune system deficits that occur earlier and result from maltreatment that may then lead to these later consequences which, in turn, lead to residence in more disadvantaged neighborhoods. Over time, these relationships may become reciprocal where lead exposure may increase the risk of maltreatment [59].

Our findings also indicate that objectively defined neighborhood disadvantage and lower individual level socio-economic status in adulthood are associated with blood lead levels later in adulthood. Neighborhood poverty has been associated with poorer health among residents [60, 61] and blood lead levels have been associated with a range of negative effects on health [62, 63]. The links among childhood maltreatment, neighborhood characteristics and individual level SES in adulthood, and dust and blood lead levels observed in this study have important implications for health research. First, it is striking that these differences in dust lead levels are manifest 30 years after these documented childhood experiences. Second, this work crosses disciplines, which in its ability to connect individual experiences of childhood maltreatment with census level neighborhood factors and rigorously measured lead dust and blood levels, elucidates the multi-systemic effects of childhood maltreatment on economics, environmental hazards and health. Targeted interventions are needed to prevent additional negative consequences to maltreated children who are already at risk and to their offspring who may be at increased risk.
